# Simulating heterogeneous populations using Boolean models

**DOI:** 10.1186/s12918-018-0591-9

**Published:** 2018-06-07

**Authors:** Brian C. Ross, Mayla Boguslav, Holly Weeks, James C. Costello

**Affiliations:** 1 Computational Bioscience Program, University of Colorado Anschutz Medical Campus, 12801 E. 17th Ave., Aurora, CO, 80045 USA; 2 Department of Pharmacology, University of Colorado Anschutz Medical Campus, 12800 E. 19th Ave., Aurora, CO, 80045 USA; 3 Department of Biostatistics and Informatics, University of Colorado Anschutz Medical Campus, 13001 E. 17th Place, Aurora, CO, 80045 USA

**Keywords:** Boolean, Network model, Cell population, Simulation, Heterogeneity

## Abstract

**Background:**

Certain biological processes, such as the development of cancer and immune activation, can be controlled by rare cellular events that are difficult to capture computationally through simulations of individual cells. Information about such rare events can be gleaned from an attractor analysis, for which a variety of methods exist (in particular for Boolean models). However, explicitly simulating a defined mixed population of cells in a way that tracks even the rarest subpopulations remains an open challenge.

**Results:**

Here we show that when cellular states are described using a Boolean network model, one can exactly simulate the dynamics of non-interacting, highly heterogeneous populations directly, without having to model the various subpopulations. This strategy captures even the rarest outcomes of the model with no sampling error. Our method can incorporate heterogeneity in both cell state and, by augmenting the model, the underlying rules of the network as well (e.g., introducing loss-of-function genetic alterations). We demonstrate our method by using it to simulate a heterogeneous population of Boolean networks modeling the T-cell receptor, spanning ∼ 10^20^ distinct cellular states and mutational profiles.

**Conclusions:**

We have developed a method for using Boolean models to perform a population-level simulation, in which the population consists of non-interacting individuals existing in different states. This approach can be used even when there are far too many distinct subpopulations to model individually.

**Electronic supplementary material:**

The online version of this article (10.1186/s12918-018-0591-9) contains supplementary material, which is available to authorized users.

## Background

Computer models are widely used to predict behaviors of biological systems, generally by simulating a number of instances of a model and enumerating the observed outcomes. Comprehensive simulations of single cells may be realistic given recent progress in constructing elaborate cellular models [1, 2], but simulation of an entire tissue is far more difficult owing to the vast number of cells, cell types, and their interactions.

As a step towards tissue-scale modeling of cells, we consider the problem of simulating large and heterogeneous populations of *non-interacting* cells. That is, we wish to model the wide variety of cellular states and dynamics experienced by a heterogeneous population of cells in isolation from each other. The output of the approach we propose will be the frequency with which certain events happen over time in a large population. This result could be obtained by averaging a large number of traditional single-cell simulations spanning the entire population, but in extremely heterogeneous populations it becomes infeasible to simulate each distinct subpopulation, in which case the traditional recourse is to estimate the population statistics by Monte Carlo (random sampling) [3]. By design, the basic random sampling procedure captures typical outcomes of these simulations, and only rarely finds atypical occurrences. Yet some biological processes are determined by outliers [4], such as the initiation and development of cancerous cells [5–7] or immune cell clonal selection [8]. If something is known about the circumstances leading to a rare outcome, one can bias Monte Carlo to oversample that outcome and then correct for the biased sampling (a strategy known as importance sampling [9]), but oversampling inevitably introduces sampling biases.

Here we propose an alternative, exact method for simulating heterogeneous populations, which takes advantage of the observation that discrete models have a finite set of possible states. For these models, one can write the instantaneous state of some individual within the population (i.e. a single instance of a Boolean network) using a vector **b**_(*α*)_ that has an entry for every possible state, where we place a 1 in the position corresponding to state *α* of the individual and a 0 everywhere else. Assuming deterministic dynamics, the time evolution of this individual can then be written as a linear (matrix) operator *F*_*b*_, so that the time evolution to a different state *β* occurs via repeated matrix multiplications of the state vector: $\mathbf {b}_{(\beta)}(t) = F_{b}^{t-t_{0}} \mathbf {b}_{(\alpha)}(t_{0})$ where *t*_0_ and *t* are integer time steps. This approach is always possible in principle for discrete systems, even when it is too computationally expensive to be feasible in practice.

**Fig. 1:**
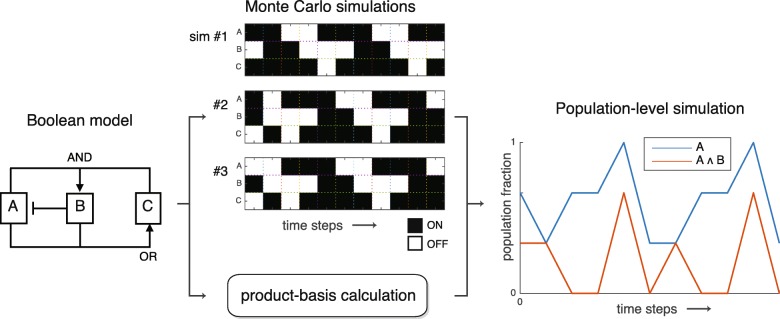
A population-level simulation. The plot shows how the population fractions displaying activation of variable *A* and variable *A*∧*B* evolve over time when the individuals in the population have heterogeneous states. No information about the substructure of the population is lost in the averaging process when one takes into account higher-order correlations (such as *A*∧*B*)

The usefulness of a linear representation is that the same equations that simulate an individual *automatically generalize to simulating arbitrary mixed populations* of individuals in different states. The basic idea, illustrated in Fig. 1, is that a population-averaged vector $\left < \mathbf {b} \right > = \sum _{\alpha } w_{\alpha } \mathbf {b}_{(\alpha)}$ evolves according to the same time evolution operator *F*_*b*_ as does a vector representing an individual, owing to the superposition property of linear systems. We will exploit this fact when we derive a time evolution operator using an algebra tailored for an individual, and then repurpose that operator to simulate mixed populations.

The obvious drawback of the linear method of time evolution is the typically huge number of states in a given system, causing both the state vector **b** and the time evolution operator *F*_*b*_ to be infeasibly large. For example, Boolean networks are a class of simple models built entirely from ON/OFF variables, yet even these models have an exponential number of states (2^*N*^ for *N* Boolean variables in the model). A linear representation of the dynamics is usually only feasible if one looks at a small subspace of the full linear space – i.e. considers only a small subset of the possible set of states of the model. However, this strategy is incompatible with our interest in simulating massively heterogeneous populations: a simulation involving *n*_*s*_ subpopulations necessarily involves at least *n*_*s*_ nonzero entries in both the state vector for the population <**b**> and in the time evolution matrix operator *F*_*b*_. Under this proportional scaling with heterogeneity, a linear representation of the system offers no computational improvement over simple enumerative simulation of each individual subpopulation.

Our proposed solution to the problem of proportional scaling with heterogeneity is to *change linear basis* from the state basis **b** to a new basis **x** in which the size of the *subspace of interest* scales independently of the heterogeneity of the mixed population. (The full spaces of **b** and **x** are necessarily of the same size.) Specifically, the goal of this paper is to introduce a linear basis for Boolean variables that we term a ‘product basis’, and give a prescription for calculating the time evolution operator in this basis. Within the Boolean framework, the product basis method is very general, applying to deterministic Boolean models [10], as well as probabilistic [11] and continuous-time [12] Boolean models in the large population limit. Note that although we assume a synchronous updating rule for all variables in the model, this is without loss of generality because an asynchronous network can be modeled as a probabilistic network with synchronous time steps [13].

By way of comparison, we note the significant advances that have been made in analyzing the possible long-term outcomes, or *attractors* (steady states and limit cycles), of Boolean models [10, 14–22]. Attractors have been found using network reduction algorithms that find simple networks encoding the long-term behavior of more complex networks [14, 16, 23], methods that solve steady states as zeros of a polynomial equation [24], SAT methods [18, 19, 25], and binary decision diagrams [20, 21, 26] (the introduction of [15] provides a review of these techniques). These techniques differ from our proposed method in that they focus on possible long-term behaviors, whereas ours gives an explicit population-averaged simulation of a defined starting population. While our method does have the ability to generate long-term dynamical equations that can be used to find attractors (see Additional file 1: Appendix 2), it differs in that it finds the attractors of a set of variables of interest, not the attractors of the complete state.

We first derive the procedure for calculating a product basis simulation to track transient and long term model behaviors. Initially we consider simulations of *individuals*, defined throughout this work as single instances of a Boolean network; then we use the same mathematics to directly simulate mixed populations of individuals in many different states. A toy example calculation is given to illustrate the mathematics. Finally, we demonstrate an application of our method by using it to simulate a large heterogeneous population of individuals whose dynamics is described by a published T-cell network [27].

## Methods

Here we consider a Boolean network model that consists of *N* variables, which updates its state at each time step using a deterministic update rule. Initially we will focus on individuals described by this model, whose instantaneous state is described by Boolean values for model variables *B*_1_,*B*_2_…*B*_*N*_ which evolve according to the rules of the model. We index these model variables using Roman letters (e.g., *i,j*,…), and use Greek letters (e.g., *α*,*β*,…) to refer to subsets of the model variables. For each possible subset of model variables *α*={*i,j*,… }, there is a unique state basis variable *b*_*α*_ (which we sometimes write as *b*_*ij*…_) and a unique product basis variable *x*_*α*_=*x*_*ij*…_, which we formally define below. We consider the state basis variables to be the components of a vector **b**, and the product basis variables to be components of vector **x**. There are 2^*N*^ subsets of all *N* Boolean model variables (including both the set *θ* of all model variables and the empty subset denoted *∅*), so both **b** and **x** are vectors of length 2^*N*^.

### **Definition 1**

Consider a Boolean model composed of a set of Boolean variables *θ*, where *B*_*i*_ represents the state of model variable *i*∈*θ*; *B*_*i*_=1 for ON and *B*_*i*_=0 for OFF. If *κ* is the set of ON variables describing the instantaneous state of some individual, then the values of the state space and product space variables describing the state of that individual, indexed by subset *α*, are defined by:

$$\begin{aligned} b_{\alpha} &= \left(\prod_{i \in \alpha} B_{i} \right) \cdot \left(\prod_{i \in (\theta \backslash \alpha)} (1 - B_{i}) \right) &= \left\{ \begin{array}{ll} 1 & \textrm{if }\alpha = \kappa\\ 0 & \text{otherwise} \end{array}\right.\\ x_{\alpha} &= \prod_{i \in \alpha} B_{i} &= \left\{ \begin{array}{ll} 1 & \textrm{if }\alpha \subseteq \kappa\\ 0 & \text{otherwise}. \end{array}\right. \end{aligned} $$

The empty product *x*_*∅*_ equals 1. The fact that each *x*_*α*_ is a simple product of model variables motivates the terminology ‘product basis’. As shown in Additional file 1: Appendix 1, a product basis vector **x** is a fully equivalent representation of a mixed population to the state basis vector **b**.

### Simulations of mixed populations

We build our simulations by selecting a set of product basis variables of interest and associating an update rule *f*_*α*_ with each variable *x*_*α*_ so that *x*_*α*_(*t*+1)=*f*_*α*_(**x**(*t*)) (the exception being the case of continuous-time Boolean networks in which *f*_*α*_=*dx*_*α*_/*dt*, but we will treat those separately later). We construct the simulation in two steps. The first step is to build the single-index update rules *f*_*i*_ (i.e. *α*={*i*}) over all model variables *i*, by enumeration of their input states. The second step is to build certain multi-index update rules *f*_*ij*…_ as needed until the system of equations closes (i.e. until we have solved for each *f*_*α*_ corresponding to a variable *x*_*α*_ appearing in another time evolution equation *f*_*β*_). To begin with, we show how to build the change-of-basis operator *T* that converts state space basis vectors to product space basis vectors through the formula **x**=*T*·**b**.

#### **Algorithm 1**

(Constructing a change-of-basis matrix) Consider any set *θ*^′^ containing *n*≤*N* of the model variables, for which *κ* and *α* denote subsets. Define *T*^(*n*)^ as the change-of-basis matrix that converts a length- 2^*n*^**b** vector indexed by *κ* to a length- 2^*n*^**x** vector indexed by *α*, and let $T^{(n)}_{\alpha \kappa }$ denote the matrix element projecting *b*_*κ*_ onto *x*_*α*_. We construct *T*^(*n*)^ by assigning a 1 to each matrix element $T^{(n)}_{\alpha \kappa }$ for which *α*⊆*κ*, and 0 to all other elements.

#### *Proof*

Consider the state vector **b**_(*κ*)_ whose entries are all 0 except for a 1 in the position corresponding to state *κ*, which describes an individual in state *κ*. The product basis representation **x** of this individual is found by multiplying $x_{\alpha } = \sum _{\gamma \subseteq \theta '} T^{(n)}_{\alpha \gamma } b_{\gamma } = T^{(n)}_{\alpha \kappa }$; thus **x** equals the column of the change-of-basis matrix that multiplies *b*_*κ*_. Using Definition 1, the value of the product basis variable *x*_*α*_, corresponding to matrix element $T^{(n)}_{\alpha \kappa }$, is 1 if *α*⊆*κ* and 0 otherwise. □

We can now provide a procedure for calculating the single variable update rules *f*_*i*_. To do so, we consider only the relatively small subset of variable *i*’s inputs, rather than the full set of model variables. We use a superscript [ *i*] to denote quantities pertaining to the input subset; thus we define *N*^[*i*]^ as the number of inputs to model variable *i*, *θ*^[*i*]^ as the set of those input variables, **b**^[*i*]^={*b*_*ρ*_∣*ρ*⊆*θ*^[*i*]^} as the state space of input variables, and **x**^[*i*]^={*x*_*ρ*_∣*ρ*⊆*θ*^[*i*]^} as the corresponding product space. In biological networks, *N*^[*i*]^ is usually small enough that we can explicitly write the change-of-basis operator $T^{\left (N^{[i]}\right)}$ in this space using Algorithm 1.

#### **Algorithm 2**

(Computing *f*_*i*_) Define **k**^[*i*]^ as a row vector such that $k_{\alpha }^{[i]}$ is 1 if the pattern of Boolean inputs $b_{\alpha }^{[i]}$ produces a 1 in output variable *i*, and 0 otherwise. Then $f_{i} = \mathbf {k}^{[i]} \left (T^{\left (N^{[i]}\right)} \right)^{-1} \mathbf {x}^{[i]}$, which is a linear equation in **x**^[*i*]^⊆**x**.

#### *Proof*

By definition *f*_*i*_=**k**^[*i*]^·**b**^[*i*]^, as this expression reproduces the output rule. Using the fact that $T^{\left (N^{[i]}\right)}$ is invertible (proved in Additional file 1: Appendix 1), we write $f_{i} = \mathbf {k}^{[i]} \left (T^{\left (N^{[i]}\right)} \right)^{-1} T^{\left (N^{[i]}\right)} \mathbf {b}^{[i]}$ and note that $T^{(N^{[i]})} \mathbf {b}^{[i]} = \mathbf {x}^{[i]}$, which proves the formula. □

Using the set of single-index *f*_*i*_, one can compute the linear time evolution function of any multi-index product basis variable *f*_*ij*…_ using the following method.

#### **Algorithm 3**

(Computing *f*_*ij*…_) First compute *f*_*α*_ for *α*={*i,j*,… } as *f*_*α*_←*f*_*i*_(**x**)·*f*_*j*_(**x**)·… (expressed in terms of **x**-basis variables). Next, distribute each term inside the product, so that *f*_*α*_ is a weighted sum of products of **x**-basis variables, and replace each nonlinear product of terms *x*_*β*_·*x*_*γ*_·… appearing inside *f*_*α*_ with the product basis variable *x*_*μ*_ where *μ*=*β*∪*γ*∪⋯. This gives an expression for *f*_*α*_ that is linear in **x**.

#### *Proof*

First we show that *f*_*α*_=*f*_*ij*…_ equals the product *f*_*i*_·*f*_*j*_·…:

$$\begin{array}{*{20}l} f_{ij\dots}(t) &= x_{ij\dots}(t+1)\\ &= x_{i}(t+1) \cdot x_{j}(t+1) \cdot \dots\\ &= f_{i}(t) \cdot f_{j}(t) \cdot \dots\\ &= f_{i} \cdot f_{j} \cdot \ldots \end{array} $$

where the last line emphasizes that there is no time dependence in *f*_*ij*…_.

The second step is to show that each *x*_*β*_·*x*_*γ*_·… equals *x*_*β*∪*γ*∪…_. Let *k,l*,… be the elements of *μ*=*β*∪*γ*∪…; then $x_{\beta } \cdot x_{\gamma } \cdot \ldots = x_{k}^{p_{k}} \cdot x_{l}^{p_{l}} \cdot \dots $ where *p*_*k*_,*p*_*l*_,⋯≥1 are the respective number of times *k,l*,… appear in *β*,*γ*,⋯. Since all *x*_*i*_ are Boolean variables, we have $x_{i}^{p} = x_{i}$ for any *p*≥1. Thus *x*_*β*_·*x*_*γ*_·…=*x*_*k*_·*x*_*l*_·…=*x*_*μ*_. □

Using Algorithms 2 and 3, we can give the full procedure for building a simulation that time evolves any arbitrary set of product basis variables of interest describing some individual modeled by the Boolean rules. We denote the set of product variables of interest as *Ω*_0_; note that each element of *Ω*_0_ is itself a set of indices over model variables. Our algorithm constructs *f*_*α*_ for each *α*∈*Ω*_0_, then additional *f*_*β*_ to evolve each *x*_*β*_ that appears in the formula for *f*_*α*_, etc. until the equations form a closed system (i.e. there is an update rule for every product basis variable appearing in the system of equations).

#### **Algorithm 4**

(Building a product basis simulation) First compute the full set of single-index *f*_*i*_ using Algorithm *2*. Next, initialize the total set of required product basis variables *Ω*←*Ω*_0_, and the set of product basis variables whose dynamics we have solved *Ω*_*S*_←*∅*. Finally, iteratively solve *f*_*α*_ for each *α*∈*Ω*∖*Ω*_*S*_ using Algorithm *3*, while updating *Ω*_*S*_←*Ω*_*S*_∪{*α*} and *Ω*←*Ω*∪{*μ*} for each variable *x*_*μ*_ appearing in the formula for *f*_*α*_. Iteration continues until *Ω*_*S*_=*Ω*.

#### *Proof*

We first note that *Ω* eventually converges to a finite set owing to the fact that the number of variables |*θ*| is finite, and that *Ω*⊆*θ* never loses elements of *θ* at each iterative step. Since a) *Ω*_*S*_⊆*Ω*, b) *Ω*_*S*_ accumulates one term in *Ω*∖*Ω*_*S*_ at each iterative step, and c) *Ω* converges, the algorithm always ends in a finite number of steps. When the algorithm terminates, the set of solved variables *Ω*_*S*_ equals the set of variables *Ω* appearing in the equations, so the resulting system of equations is closed. □

Writing our final closed system of linear equations as a square matrix *F*, we have a very simple update rule for simulations using the product basis variables: **x**(*t*+1)=*F*·**x**(*t*).

Our final step is to generalize to a mixed-population simulation.

#### **Definition 2**

Define a population-level state vector <**x**> as a population-weighted linear combination of the state vectors of the subpopulations **x**_(*α*)_:

1 $$\begin{array}{*{20}l} \left< \mathbf{x} \right>(t) &:= \sum_{\alpha} w_{\alpha} \mathbf{x}_{(\alpha)}(t)  \end{array} $$

where the weighting factors *w*_*α*_ are proportional to the occurrence of subpopulations *α* in the overall mixed population.

Note that we are free to choose the normalization constant $W = \sum _{\alpha } w_{\alpha }$. In all of our examples, we will choose *W*=1, leading to the interpretation that *w*_*α*_ is the fraction of the population in state *α*. Irrespective of the choice of *W*, this representation can be used to evolve mixed populations over time using our existing operators.

#### **Claim 1**

Each element <*x*_*α*_> of the vector <**x**> is proportional to the overall occurrence of individuals having *x*_*α*_=1 in the mixed population. For *W*=1, <*x*_*α*_> is the fraction of the population having *x*_*α*_=1.

#### *Proof*

Equation 1 is the definition of a weighted average of **x**, whose weighting factors **w** are proportional to the population fraction. □

#### **Claim 2**

Our linear operator *F* that evolves any arbitrary state of an individual **x**_*α*_ over time also correctly evolves the state of any arbitrary mixed population <**x**> over time.

#### *Proof*

This Claim follows from the fact that *F* commutes with the sum over subpopulations:

2 $$\begin{array}{*{20}l} \left< \mathbf{x} \right>(t) &= \sum_{\alpha} w_{\alpha} F^{t-t_{0}} \mathbf{x}_{(\alpha)}(t_{0})\\ &= F^{t-t_{0}} \sum_{\alpha} w_{\alpha} \mathbf{x}_{(\alpha)}(t_{0})\\ &= F^{t-t_{0}} \left< \mathbf{x} \right>(t_{0}). \end{array} $$

□

Thus, the time evolution operator *F* produced by Algorithm 4 correctly evolves the mean occurrence of variables <**x**> over time in any mixed population, *despite the fact that this operator was derived for an* **x** *that describes an individual* (notably in assuming that each *x*_*α*_ is Boolean).

#### Example 1: building equations

**Fig. 2:**
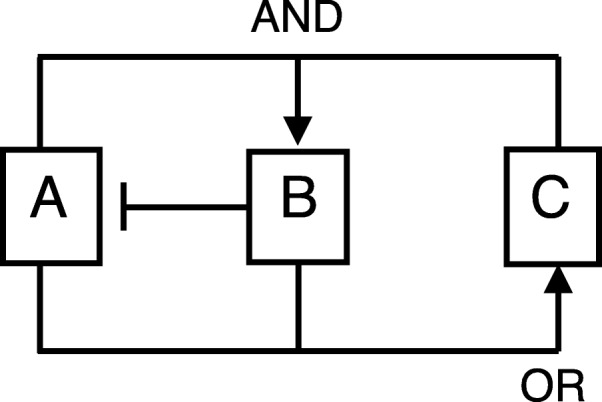
A 3-node Boolean network. The network used in Example 1. Arrows indicate the update rules for each variable: for example if either A or B is ON at time *t* then C will be ON at time *t*+1; otherwise C will be OFF

Consider the model shown in Fig. 2, whose Boolean model variables update according to the following rules:

$$\begin{array}{*{20}l} A(t+1) &= \overline{B(t)}\\ B(t+1) &= A(t) \land C(t)\\ C(t+1) &= A(t) \lor B(t). \end{array} $$

To build a product basis simulation, we first compute the change-of-basis matrices that will be used to compute the single-variable update rules *f*_*A*_, *f*_*B*_ and *f*_*C*_. Variable *A* takes input from the single variable *B*, so calculating *f*_*A*_ requires the change-of-basis matrix *T*^(1)^. Ordering the elements by the subscripts (*∅,B*) respectively, and applying Algorithm 1, we obtain

$$\begin{array}{*{20}l} T^{(1)} &= \left[ \begin{array}{cc} 1 & 1\\ 0 & 1\\ \end{array} \right]. \end{array} $$

Variables *B* and *C* each take input from two variables. Ordering *B*’s input states (*∅*,*A,C,AC*), and *C*’s input states (*∅*,*A,B,AB*), we find that both *f*_*B*_ and *f*_*C*_ are computed using

$$\begin{array}{*{20}l} T^{(2)} &= \left[ \begin{array}{cccc} 1 & 1 & 1 & 1\\ 0 & 1 & 0 & 1\\ 0 & 0 & 1 & 1\\ 0 & 0 & 0 & 1\\ \end{array} \right]. \end{array} $$

Next, we build the single-index update functions. Variable *A* takes input only from variable *B*, so the possible patterns of active inputs are (*∅*,*B*)^⊤^, corresponding to the state basis variables $\left (b^{[A]}_{\emptyset }, b^{[A]}_{B}\right)^{\top }$. The respective outputs are (1,0)=**k**^[*A*]^ due to the NOT gate, from which we can immediately calculate *f*_*A*_ using Algorithm 2:

3 $$\begin{array}{*{20}l} f_{A} &= \mathbf{k}^{[A]} \left(T^{(1)} \right)^{-1} \left[ \begin{array}{c} x_{\emptyset}\\ x_{B} \end{array} \right]\\ &= \left[ \begin{array}{cc} 1 & 0 \end{array} \right] \left[ \begin{array}{cc} 1 & -1\\ 0 & 1 \end{array} \right] \left[ \begin{array}{c} x_{\emptyset}\\ x_{B} \end{array} \right]\\ &= x_{\emptyset} - x_{B}. \end{array} $$

In the same way we find that the input patterns $\left (\emptyset, b^{[B]}_{A}, b^{[B]}_{C}, b^{[B]}_{AC}\right)^{\top }$ to variable *B* lead to outputs **k**^[*B*]^=(0,0,0,1), and the input patterns $\left (\emptyset, b^{[C]}_{A}, b^{[C]}_{B}, b^{[C]}_{AB}\right)^{\top }$ to variable *C* lead to outputs **k**^[*C*]^=(0,1,1,1). Using these together with *T*^(2)^ we compute:

4 $$\begin{array}{*{20}l} f_{B} &= \left[ \begin{array}{cccc} 0 & 0 & 0 & 1 \end{array} \right] \left[ \begin{array}{cccc} 1 & -1 & -1 & 1\\ 0 & 1 & 0 & -1\\ 0 & 0 & 1 & -1\\ 0 & 0 & 0 & 1\\ \end{array} \right] \left[ \begin{array}{c} x_{\emptyset}\\ x_{A}\\ x_{C}\\ x_{AC} \end{array} \right]\\ &= x_{AC}\\ f_{C} &= \left[ \begin{array}{cccc} 0 & 1 & 1 & 1 \end{array} \right] \left[ \begin{array}{cccc} 1 & -1 & -1 & 1\\ 0 & 1 & 0 & -1\\ 0 & 0 & 1 & -1\\ 0 & 0 & 0 & 1\\ \end{array} \right] \left[ \begin{array}{c} x_{\emptyset}\\ x_{A}\\ x_{B}\\ x_{AB} \end{array} \right]\\ &= x_{A} + x_{B} - x_{AB}. \end{array} $$

Having built the single-index update functions, we can now derive a linear system that tracks the time evolution of any set of product variables that we aim to follow. Suppose we wish to follow the time evolution of variable *x*_*A*_, corresponding to the activity (or mean activity at the population level) of the Boolean model variable *B*_*A*_. The immediate equation for this purpose is *f*_*A*_, which we already derived (Eq. 3), but since it involves *x*_*B*_ and *x*_*∅*_, our simulation must also track those variables through time using Eq. 4 along with

5 $$\begin{array}{*{20}l} f_{\emptyset} &= x_{\emptyset}. \end{array} $$

Equation 4 requires that we track a new multi-index variable *x*_*AC*_, requiring us to solve *f*_*AC*_ using Algorithm 3:

6 $$\begin{array}{*{20}l} f_{AC} &= (1-x_{B}) \cdot (x_{A} + x_{B} - x_{AB})\\ &= x_{A} + x_{B} - x_{AB} - x_{AB} - x_{B} + x_{AB}\\ &= x_{A} - x_{AB}. \end{array} $$

We continue the process of identifying new variables and solving for their update functions until the equations form a closed system:

7 $$\begin{array}{*{20}l} f_{AB} &= (1-x_{B}) \cdot (x_{AC})\\ &= x_{AC} - x_{ABC} \end{array} $$

8 $$\begin{array}{*{20}l} f_{ABC} &= (1-x_{B}) \cdot (x_{AC}) \cdot (x_{A} + x_{B} - x_{AB})\\ &= x_{AC} - x_{ABC}. \end{array} $$

Equations 3-8, together with initial values for *x*_*A*_, *x*_*B*_, *x*_*AC*_, *x*_*AB*_ and *x*_*ABC*_, describe the time evolution of these quantities in an individual Boolean network as a sequence of 0s and 1s in each variable. The final step is to reinterpret these equations as describing the dynamics of a mixed population, formally by taking the mean of both sides of each equation:

$$\begin{array}{*{20}l} f_{\left< A \right>} &= \left< x_{\emptyset} \right> - \left< x_{B} \right>\qquad\qquad\qquad\qquad\qquad\quad \text{(3{{b}})}\\ f_{\left< B \right>} &= \left< x_{AC} \right>\qquad\qquad\qquad\qquad\qquad\qquad\quad\;\, \text{(4{b})}\\ f_{\left< \emptyset \right>} &= \left< x_{\emptyset} \right>\qquad\qquad\qquad\qquad\qquad\qquad\qquad \text{(5{b})}\\ f_{\left< AC \right>} &= \left< x_{A} \right> - \left< x_{AB} \right>\qquad\qquad\qquad\qquad\qquad\;\; \text{(6{b})}\\ f_{\left< AB \right>} &= \left< x_{AC} \right> - \left< x_{ABC} \right>\qquad\qquad\qquad\qquad\quad\;\; \text{(7{b})}\\ f_{\left< ABC \right>} &= \left< x_{AC} \right> - \left< x_{ABC} \right>.\qquad\qquad\qquad\qquad\quad\;\, \text({8{b})} \end{array} $$

The angle-brackets denote an average, and we have used the notation <*x*_*i*_(*t*+1)>=*f*_<*i*>_. Per Claim 2, the linear equations are unaffected by the averaging process, so the same equations used to derive the dynamics of an individual also describe the mean values of those same variables in a mixed population. Whereas the product basis variables take on binary values for an individual, the population-averaged variables are real-valued on the interval [ 0,1] (using our recommended normalization in Claim 1). For example, we would set <*x*_*A*_>=0.4 if gene *A* is ON in 40% of the population.

**Probabilistic and asynchronous Boolean networks**

The product basis method can be applied to probabilistic Boolean networks (PBNs) [11, 28], in which several state transitions are possible at each time step with different probabilities. As we will show, our algorithm works in the *large-population limit* for which time evolution of the average state <**x**> is essentially deterministic, despite the fact that each individual PBN is stochastic.

Applying our method to PBNs requires that we reinterpret the meaning of the time evolution equations. For an individual we write:

9 $$\begin{array}{*{20}l} p(\mathbf{x}(t+1)) = F \cdot \mathbf{x}(t) \end{array} $$

where *p*(·) denotes the probability of an outcome. The product basis method still works with this new interpretation of the time evolution operator *F*, although we note several changes to the logic. First, in Algorithm 2 we generalize each **k**^[*i*]^ to be a vector of likelihoods that each respective input pattern produces a 1 in the output variable, so that *f*_*i*_=**k**^[*i*]^·**b**^[*i*]^ as before. Second, the multiplication rule used in Algorithm 3 still holds if updated to read $p_{x_{ij\dots }} = p(x_{i}) p(x_{j}) \dots $, owing to the independence of outcomes *p*(*x*_*i*_), *p*(*x*_*j*_), etc. Third, we point out that although *p*(**x**) is real-valued, the state of an individual **x** is still binary, so $x_{\alpha }^{p \ge 1} = x_{\alpha }$ as before (Algorithm 3).

Despite the fact that our algorithm produces valid product basis equations of the form of Eq. 9, the resulting linear system of equations does not form a closed system, simply because the left-hand side uses different variables than the right (*p*(**x**) versus **x**). Our method therefore cannot be used to simulate an individual instance of a PBN. However, by averaging both sides and taking the large-population limit so that *p*(**x**)→<**x**>, the system closes and reproduces Eq. 2. Thus, our system of equations accurately tracks the deterministic dynamics of arbitrary mixed populations of PBNs in the infinite-population limit, despite being unable to model stochastic individuals.

Large populations of asynchronous networks behave identically to large populations of PBNs [13] if we define a uniform time step: the probabilities of the various possible updates over that time step give the state transition weights in the corresponding synchronous PBN. If this time step is small enough, then the likelihood of two causally-connected asynchronous updates happening in the same step is small, and in this limit the local update rules for a PBN accurately model the asynchronous network. Therefore our analysis also applies to large populations of asynchronous networks for small time steps.

**Continuous-time Boolean networks**

Probabilistic networks give one way to incorporate rate information into our model; another way is to work in continuous time using differential equations [12]: *f*_*A*_=*dx*_*A*_/*dt*. The differential form does require one change in our method: the rate of change of a higher-order variable is found by using the product rule of derivatives. Whereas under a discrete update *f*_*ABC*…_ is the product *f*_*A*_·*f*_*B*_·*f*_*C*_·…, for the differential case we compute:

10 $$\begin{array}{*{20}l} f_{ABC\dots} &= \frac{d}{dt} \left(x_{A} x_{B} x_{C} \dots \right)\\ &= x_{B} x_{C} \dots f_{A} + x_{A} x_{C} \dots f_{B} + \cdots. \end{array} $$

Also, under discrete updates, the trivial function is *f*_*∅*_=1, but with differential updates it is *f*_*∅*_=0.

## Results

**Product basis equations recapitulate Monte Carlo simulations**

We tested the product basis method using 10^4^ randomly generated 10-node deterministic networks, where each node took input from 1-4 randomly selected nodes using randomly generated logic rules (∼ 25 edges per network). For each network, we ran 100 Monte Carlo individual simulations using a random ensemble of initial states (equal probability for all states), and compared the population average of model variables with a product basis simulation using the same starting population. In each case, the product basis simulation reproduced the average of the Monte Carlo simulations. Next, we ran 10^4^ tests of probabilistic networks (PBNs), again using 100 Monte Carlo runs per test. In order to generate realistic PBNs, we augmented the original time evolution functions *f*_*i*_ with random rate parameters *r*_*i*_ on the interval 0≤*r*_*i*_≤1, leading to the equations *fi*′←(1−*r*_*i*_)*x*_*i*_+*r*_*i*_*f*_*i*_ for 0≤*r*_*i*_≤1. The rate parameters control the fraction of an update taken over a time step: a small value of *r*_*i*_ means *x*_*i*_ changes little over a single time step (reflecting a slow process), whereas *r*_*i*_=1 reproduces the update rule of a deterministic network. As we found with the discrete Boolean networks, the equations of the product basis method reproduced the results of Monte Carlo simulations, this time to within sampling error, which is proportional to $ 1 / \sqrt {n_{runs}}$.

Finally, we tested 2.5×10^3^ continuous-time networks generated using the same rule, and again found complete agreement with Monte Carlo. We tested fewer continuous-time networks because they took considerably longer to run, owing to the need to integrate differential equations (for both the product basis simulations and their Monte Carlo comparisons).

**Product basis method can simulate highly heterogeneous populations beyond the scope of Monte Carlo**

**Fig. 3:**
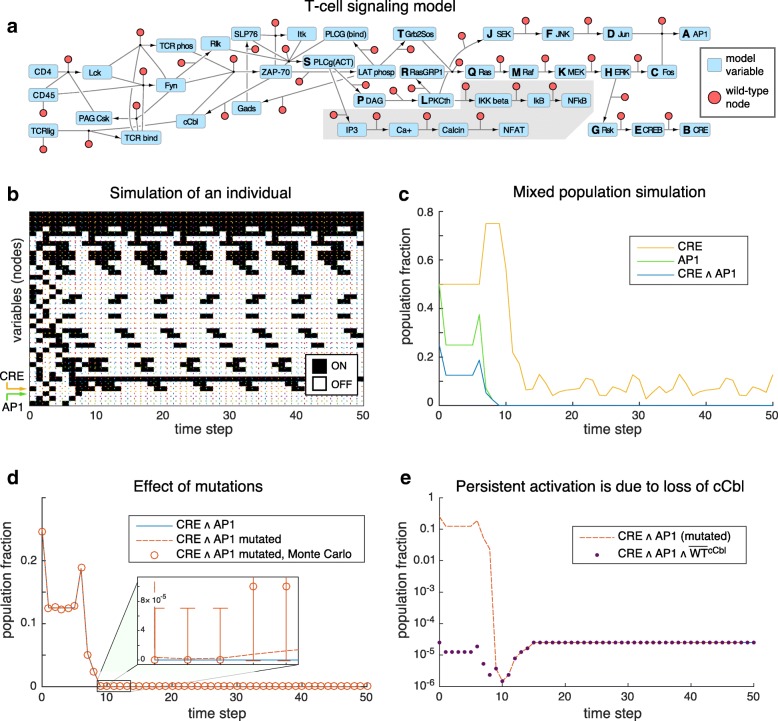
Simulation of a heterogeneous population of T-cell networks. **a** Boolean model of a T-cell activation, introduced in [27]. Model variables correspond to blue nodes; red nodes are introduced to describe loss-of-function alterations of the network. Model variables used in the example equations in the text are given boldface letters corresponding to their subscripts. **b** Time evolution of one individual modeled by the T-cell network, starting from a random initial state. White/black rectangles signify OFF/ON Boolean states. **c** Time evolution of the population fraction having activated CRE elements and/or expressing the transcription factor AP1 in a heterogeneous population of T-cell networks, computed using a product basis calculation. The heterogeneous population begins at *t*=0 as a uniform mixture of all possible 2^33^≈10^10^ initial states of the upstream portion of the model. **d** The effect of a 10^−4^ knock-out mutation rate per gene in the heterogeneous population. Monte Carlo, but not the product basis calculation, required this high rate of mutations in order to detect persistent coactivation of CRE and AP1. **e** The co-occurrence of CRE activation and AP1 expression in mutated networks shown on a log_10_ scale (dotted red line), compared with the amount of this coexpression coincident with mutated cCbl (purple dots). The mutated fraction was computed by subtracting the time series of CRE ∧ AP1 ∧ WT-cCbl from the time series of CRE ∧ AP1

To demonstrate the product basis method on very heterogeneous populations, we applied it to the T-cell activation network described in Figure 10 and Table 2 of [27], which we have re-illustrated in Fig. 3a. The T-cell network is a deterministic, 40-node network with multiple feedback loops and whose attractors include both steady states and limit cycles. For demonstration purposes, we show a traditional Monte Carlo simulation of an *individual* modeled by the T-cell network in Fig. 3b, obtained by choosing an initial Boolean state and applying the model rules over each successive time step. This particular simulation shows transient (nonrepeating) behavior for the first 10 time steps, leading to a limit cycle with a repeating period of 6 time steps.

Next, we performed a population-level simulation using the product basis method. We opted to track the time evolution of three downstream variables in the model: cyclic-AMP response element (CRE) mediated gene activation, the AP1 (Activating protein 1) transcription factor, and their co-occurrence whose variable we label CRE ∧ AP1. To perform this simulation, we initialized Algorithm 4 using product basis variables representing these quantities:

$$\begin{array}{*{20}l} \Omega_{0} = \{ x_{\mathrm{A}}, x_{\mathrm{B}}, x_{\text{AB}} \}. \end{array} $$

Here we use *A* to denote AP1 and *B* to denote CRE (boldface letters in Fig. 3a). CRE and AP1 were chosen for demonstration purposes because they lie at the downstream ends of two separate branches of the network, which taken together are influenced by 33 of the 40 variables in the model. The co-occurrence variable was not redundant: although CRE ∧ AP1 is simply the product of the Boolean states of CRE and AP1 in an individual, this is *not* true at the population level. For example if the levels of CRE and AP1 are both 0.5, then the level of CRE ∧ AP1 could equal 0.25 if CRE and AP1 states are uncorrelated in the population, 0 if they are perfectly anticorrelated, 0.5 if they are perfectly correlated, or any other value on the interval [ 0,0.5].

Next, we ran the product basis method to generate the time evolution equations. The resulting system of equations included our three variables of interest, along with 1049 other variables that were added in the process of closing the system of equations; accordingly, the full time evolution operator was a 1052-square matrix. A typical ‘ladder’ of time evolution equations governing a quantity of interest, in this case the mean activity level of CRE ∧ AP1 in the population denoted by variable *x*_*AB*_, begins with the equations:

$$\begin{aligned} f_{AB} &= x_{CDE}\\[-3pt] f_{CDE} &= x_{FGH}\\[-3pt] f_{FGH} &= x_{HJK}\\[-3pt] f_{HJK} &= x_{KLM}\\[-3pt] f_{KLM} &= x_{MPQ}\\[-3pt] f_{MPQ} &= x_{QRS} + x_{QSU} - x_{QRSU}\\[-3pt] &\dots \end{aligned} $$

where *C,D*,… correspond to upstream nodes Fos, Jun, etc., as indicated in Fig. 3a. The first few equations are quite simple in the product basis because of the simple feedforward nature of the terminal portion of the network, although they get much more complicated further upstream. This simplification would not be possible in the state basis, illustrating a strength of our approach. The list of equations we show was truncated by necessity: 976 variables with their respective 976 update rules are required to specify the time evolution of CRE ∧ AP1. (The other 76 variables in the 1052-variable system are involved in specifying the time evolution of CRE or AP1 individually but not their co-occurrence.)

After generating the product basis time evolution equations, we set the model variables to an initial state representing a mixed population, then used the time evolution equations to track the population level average of each of the three variables for 50 time steps (Fig. 3c). We stress that this is an exact result, with no sampling error. The starting population we considered was a uniform mixture of all possible 2^40^ initial states of the Boolean network, but because 7 of these variables are not upstream of either CRE or AP1 (see shaded region in Fig. 3a), we consider our effective initial population size to be 2^33^≈10^10^. At this level of diversity, it would require extensive computation to reproduce our exact result by exhaustive enumeration over the initial states.

Next, we demonstrated the ability of the product basis method to analyze loss-of-function genetic alterations, encompassing copy number loss, insertions, deletions, SNPs, etc. To allow every possible genetic alteration in the population, we added a set of ‘wild-type’ variables to the network, one for each original model variable, and included the wild-type variables in the update rules using an AND operation. For example, an update rule reading [ *A*←*B* ∨ *C*] became [*A*←(*B* ∨ *C*) ∧ *A*^WT^]. Note that the presence of the wild-type variables effectively doubles the size of the network and thus vastly increases the heterogeneity, which is determined by both the number of activation states of the original variables and the number of mutational profiles, in total spanning the order of 10^20^ different subpopulations, compared to 10^10^ without genetic alterations. Enumeration over the initial states is impossible at this level of diversity, and the traditional solution is a sparse sampling method such as Monte Carlo, which lacks the ability to resolve very rare subpopulations.

Despite the massive heterogeneity of the genetically altered population, we were successfully able to use our product basis method to construct the exact time evolution equations for CRE, AP1 and CRE ∧ AP1 as before. This time we focused on CRE ∧ AP1 (i.e. *Ω*_0_={*x*_*AB*_}) to more clearly show the comparison with Monte Carlo. Solving for the time evolution of CRE ∧ AP1 involved the addition of 18,957 variables beyond *x*_*AB*_, requiring a much larger time evolution operator than before (a 18,958^2^ element matrix). These new time evolution equations depend on the genetic alteration state of the system through the presence of wild-type variable subscripts, as we illustrate below by listing the first few equations governing the dynamics of *x*_*AB*_. For brevity, these equations use a tilde to denote the wild-type variables: for example $x_{\tilde {A}}$ gives the state of the wild-type variable *A*^WT^.

$$\begin{array}{*{20}l} f_{AB} &= x_{CDE\tilde{A}\tilde{B}}\\ f_{CDE\tilde{A}\tilde{B}} &= x_{FGH\tilde{A}\tilde{B}\tilde{C}\tilde{D}\tilde{E}}\\ f_{FGH\tilde{A}\tilde{B}\tilde{C}\tilde{D}\tilde{E}} &= x_{HJK\tilde{A}\tilde{B}\tilde{C}\tilde{D}\tilde{E}\tilde{F}\tilde{G}\tilde{H}}\\ f_{HJK\tilde{A}\tilde{B}\tilde{C}\tilde{D}\tilde{E}\tilde{F}\tilde{G}\tilde{H}} &= x_{KLM\tilde{A}\tilde{B}\tilde{C}\tilde{D}\tilde{E}\tilde{F}\tilde{G}\tilde{H}\tilde{J}\tilde{K}}\\ f_{KLM\tilde{A}\tilde{B}\tilde{C}\tilde{D}\tilde{E}\tilde{F}\tilde{G}\tilde{H}\tilde{J}\tilde{K}} &= x_{MPQ\tilde{A}\tilde{B}\tilde{C}\tilde{D}\tilde{E}\tilde{F}\tilde{G}\tilde{H}\tilde{J}\tilde{K}\tilde{L}\tilde{M}}\\ f_{MPQ\tilde{A}\tilde{B}\tilde{C}\tilde{D}\tilde{E}\tilde{F}\tilde{G}\tilde{H}\tilde{J}\tilde{K}\tilde{L}\tilde{M}} &= x_{QRS\tilde{A}\tilde{B}\tilde{C}\tilde{D}\tilde{E}\tilde{F}\tilde{G}\tilde{H}\tilde{J}\tilde{K}\tilde{L}\tilde{M}\tilde{P}\tilde{Q}}\\ &\hspace{.3in} + x_{QSU\tilde{A}\tilde{B}\tilde{C}\tilde{D}\tilde{E}\tilde{F}\tilde{G}\tilde{H}\tilde{J}\tilde{K}\tilde{L}\tilde{M}\tilde{P}\tilde{Q}}\\ &\hspace{.3in} - x_{QRSU\tilde{A}\tilde{B}\tilde{C}\tilde{D}\tilde{E}\tilde{F}\tilde{G}\tilde{H}\tilde{J}\tilde{K}\tilde{L}\tilde{M}\tilde{P}\tilde{Q}}\\ &\dots \end{array} $$

In order to explicitly simulate a genetically altered population, we chose an initial state containing each possible combination of loss-of-function alterations at a 0.01% mutation rate per variable (roughly the highest possible rate of homozygous losses-of-function given a 1%-per-gene-locus mutation rate [29]), as well as the uniform mixture of each activation state that we considered with the previous simulation (adjusted so that mutated genes always began OFF). From this initial state, we again followed the exact time course of the CRE ∧ AP1 population fraction, and compared it to our original wild-type result (Fig. 3d). Notably, a small fraction of the population 0.0025*%* reached a steady state showing both CRE activation and AP1 expression. We also validated the result (to within statistical error) using Monte Carlo, although as shown in Fig. 3d, Monte Carlo was only useful for comparing the early transient behavior, not the rare subpopulations that persisted at late time at levels as low as 0.00015*%*.

Finally, we examined the mutations leading to CRE ∧ AP1 coexpression. We hypothesized that this was due to loss of cCbl in the recurrent core of this network. We tested this hypothesis by generating the time course of the three-way co-occurrence of CRE ∧ AP1 ∧ WT-cCbl, where WT denotes the respective wild-type variable. This final time series dropped to exactly zero at steady state, indicating that loss of cCbl is necessary for persistent CRE ∧ AP1 coexpression (see Fig. 3e), and that absolutely no other set of loss-of-function alterations could recapitulate this phenotype.

Our results from the T-cell network demonstrate several important aspects of our method. First, we are able to simulate extremely heterogeneous populations, involving far more subpopulations than could be analyzed individually. Second, although our method only deals with heterogeneity in the states of the Boolean variables, we can still simulate a genetically-heterogeneous population by augmenting the Boolean network with wild-type variables. Third, we can exactly model subpopulations that are present at very low levels, which are difficult to resolve by random sampling (see the error bars in Fig. 3d). For example, the contribution of each triple-gene-loss was factored in even though a given triple-gene-loss was present in only 10^−12^ of the population. While one might artificially raise the Monte Carlo mutation rate to oversample the mutations [9], this has the disadvantage of overweighting the effect of multiple loss-of-function genes, even though realistic evolutionary paths take one or very few genetic losses at a time [30]. In contrast, our exact result is dominated by the evolutionarily-accessible subpopulations that are closest to wild-type.

**Code availability** The MATLAB scripts used to test the method and run the T-cell example are named testRandomNetworks.m and tCellActivationEx.m respectively, and are available for download at https://github.com/CostelloLab/ProductBasis. The equation generating process for Fig. 3c and d took ∼ 3 and ∼ 300 seconds respectively using our code (written in MATLAB R2015b 8.6.0.267246, running on a 2.6GHZ Intel core i7 Mac with OS 10.9.5). The Monte Carlo comparison in Fig. 3d (*n*_*runs*_=10^4^) took ∼ 140 seconds.

## Discussion

Our product basis method allows the direct simulation of highly heterogeneous populations, including the transient processes that are ignored by Boolean attractor analyses. This strategy can be applied to any system involving heterogeneous populations, as long as the individuals in a population can be modeled using Boolean logic. Our approach can be used to follow single variables of the system over time, as well as the correlations between these variables that are both necessary and sufficient to fully describe the dynamics of the population. We also showed that our method, when applied to a network augmented by wild-type nodes, can effectively explore heterogeneity in the network rules in addition to heterogeneity in network state. Our example using the T-cell activation network explored perturbations leading to complete loss-of-function in network nodes. We could have also allowed gain-of-function alterations of network nodes, or the combination of loss- or gain-of-function effects on network edges by a adding wild-type node for each edge and incorporating them into the update rules. In each of our simulations, all subpopulations are exactly accounted for in the output time series, no matter how rare.

The key to our method is to write the time evolution equations as a linear system, but in a different basis than the usual state space basis. The product basis variables have several advantages over state space variables. First, ordinary descriptions of mixed populations usually correspond more closely to the product basis variables than to individual states. For example, we might specify that half the population starts with both genes *A* and *B* on, which translates into the condition *x*_*AB*_=0.5 in the product basis, but a much more complicated condition on the state basis variables (namely that the sum over all variables having both *A* and *B* in their indices equals 0.5). Another advantage is that the product basis equations often close using relatively few equations irrespective of the heterogeneity of the population, whereas the number of equations required in the state space basis increases with heterogeneity: the simulations we showed in Fig. 3 would require all 2^40^ state space variables. Thus our choice of variables is better for modeling very heterogeneous populations. Finally, as shown in Additional file 1: Appendix 2 the product basis allows for a variable factorization scheme that can help to simplify the calculation if we only care about the long-term behavior.

We acknowledge that our method can become intractable for complex networks due to the fact that construction of these simulations can be an exponential problem, depending on the complexity of the network. Fully connected Boolean networks with random logic rules will always be challenging, but we believe it should be possible to improve performance on certain network motifs, such as downstream feedback loops, that give our method difficulty. The product basis is only one of very many linear bases to choose from, and while it works well for some network topologies such as the feedforward arms of the T-cell network, other choices of basis may well perform better on other network motifs. Future work will explore this possibility. Additionally, the equation reduction method for finding attractors (Additional file 1: Appendix 2) is more an art than a science, and our future work will aim to improve this part of the calculation for typical network models (although the attractor analysis is also known to be NP-hard [31]).

## Conclusions

Molecular and phenotypic heterogeneity play major roles in such varied systems as healthy and cancerous tissues, evolution at the organism scale, and immune activation [32]. In all of these cases, rare and unexpected dynamics are difficult to capture by simulations of individuals, while pure attractor analyses may miss important aspects of the dynamics such as the transient behavior and the size of the attractive basins. We have demonstrated that the methodology outlined here can help to capture these important but elusive events.

## Additional file

Additional file 1 Appendices (PDF 299 kb)
