# Supplementary material for: Simulating heterogeneous populations using Boolean models
Source: BMC Syst Biol. 2018 Jun 7;12:64. doi: 10.1186/s12918-018-0591-9 (PMC5992775; doi:10.1186/s12918-018-0591-9)
Supplement: Supplementary file 1 — Appendices (PDF 299 kb) [file 12918_2018_591_MOESM1_ESM.pdf]

Appendices

Appendix 1: product variables form a complete and independent basis spanning the state space

Claim 3. *The change-of-basis matrix carrying* $\mathbf{b}$ *variables to* $\mathbf{x}$ *variables is an invertible transformation.*

Proof. Our proof first requires us to explicitly write the change-of-basis matrix T. We can do this using Algorithm 1 once we have chosen a particular ordering of the entries of both the $\mathbf{b}$ and $\mathbf{x}$ vectors. Here we will choose to order the entries of both $\mathbf{b}$ and $\mathbf{x}$ by binary order of the subscripts on their respective state variables. In other words, the index is given by the binary number having 1s in the positions indicated by the subscripts of each state variable: the state variable written as $b_\alpha = b_{ij\ldots}$ is the mth entry in the state space vector $\mathbf{b}$ and $x_\alpha = x_{ij\ldots}$ is the mth entry in the product space vector $\mathbf{x}$, where $m = 2^{i-1} + 2^{j-1} + \cdots + 1$, for $1 \leq \{i, j, \ldots\} \leq N$. This choice of ordering fixes a particular representation of the change-of-basis matrix. For example, for the case of three Boolean variables A, B and C, the change-of-basis matrix is

$$T^{(3)} = \begin{array}{c} \\ 1 \\ x_A \\ x_B \\ x_{AB} \\ x_C \\ x_{AC} \\ x_{BC} \\ x_{ABC} \end{array} \begin{array}{cccccccc} b_\emptyset & b_A & b_B & b_{AB} & b_C & b_{AC} & b_{BC} & b_{ABC} \\ \end{array} \left[\begin{array}{cccccccc} 1 & 1 & 1 & 1 & 1 & 1 & 1 & 1 \\ 0 & 1 & 0 & 1 & 0 & 1 & 0 & 1 \\ 0 & 0 & 1 & 1 & 0 & 0 & 1 & 1 \\ 0 & 0 & 0 & 1 & 0 & 0 & 0 & 1 \\ 0 & 0 & 0 & 0 & 1 & 1 & 1 & 1 \\ 0 & 0 & 0 & 0 & 0 & 1 & 0 & 1 \\ 0 & 0 & 0 & 0 & 0 & 0 & 1 & 1 \\ 0 & 0 & 0 & 0 & 0 & 0 & 0 & 1 \end{array} \right].$$

For notational convenience, the two lemmas below denote matrix elements using scalar indices as in T_{mp}, rather than using their corresponding set variables as in $T_{\alpha\beta}$. Likewise a product variable x_α is sometimes denoted here as x_m, referring to the position of x_α in $\mathbf{x}$ using the above representation.

Our objective is to show that the change-of-basis matrix is always upper-triangular, with 1s on the diagonal.

Lemma 1. *The* m^{th} *diagonal entry* T_{mm} *equals 1.*

Proof. T_{mm} projects b_α onto x_α for some α, so it can be written $T_{\alpha\alpha}$. Referring to algorithm 1, $\alpha \subseteq \alpha$ so this matrix element is 1. □

Lemma 2. *Every entry below the diagonal (i.e.* T_{mp} *for* $m > p$*) equals 0.*

Proof. Matrix element T_{mp} projects state basis variable $b_p = b_\beta$ onto product basis variable $x_m = x_\alpha$. By Algorithm 1 this matrix element is either 0 or 1. Assume it is a 1, implying that $\alpha \subseteq \beta$. Then $m = \sum_{i\in\alpha} 2^{i-1} + 1$ and $p = \sum_{i\in\beta} 2^{i-1} + 1 = m + \sum_{i\in(\beta\setminus\alpha)} 2^{i-1}$, implying that $p \geq m$. This contradicts the assumption that $m > p$, so $T_{mp} = 0$. □

Thus each diagonal entry of the change-of-basis matrix is 1, and the entire lower triangle is 0. Since the change-of-basis matrix is triangular, the eigenvalues are the diagonal entries, which are all 1. Therefore the determinant is 1, so the change-of-basis transformation is non-degenerate (and volume-preserving) and therefore invertible. □

Appendix 2: equation reduction for late time behavior

A mixed-population simulation may or may not be practical, depending on whether the system of linear equations closes with a manageable number of variables. One way to simplify the equations is to focus on the late-time behavior of the system, after quickly-decaying eigenmodes have dropped near zero and time evolution effectively occurs in the smaller subspace of slowly-decaying or persistent eigenmodes. In the limit where we focus on the infinite-time behavior, this subspace is simply the attractors of the system (steady states and/or limit cycles). Our general strategy will be to identify fast-decaying modes as they appear in the time evolution matrix operator F, set them to zero and use the resulting expression as a constraint that can be used to remove variables from later steps of the calculation. In order to be useful for simplifying calculations, these steps must be taken before the linear system closes – in other words, when our partially-computed update function $F_{partial}$ is still a non-square matrix.

Our strategy for finding decaying modes in $F_{partial}$ relies on a QR decomposition, which can be performed on non-square matrices. In the case of a deterministic network, all eigenvalues of the full time evolution operator F have modulus 0 or 1 implying that all decaying modes are strict linear dependencies. (*Proof*: The product space is finite, so every eigenmode λ must either decay to zero in finite time implying that $\lambda = 0$, or else repeat in Δt time steps so that $|\lambda| = 1$ and $\arg(\lambda) = 2\pi k/\Delta t$ for integer k.) The set of linearly-dependent rows in F is found by identifying (numerically) zero entries along the diagonal of the R matrix from the QR decomposition. The dependencies themselves are then calculated by solving the matrix equation $N = KD$ for polynomial coefficients K, where N is the submatrix that contains the independent rows of F and D is the submatrix that contains the dependent rows of F. For PBNs, the complication is

that decaying eigenvalues of the full system are generally nonzero, so the decaying modes are not simple linear dependencies. In order to find modes in the $m \times n$ matrix $F_{partial}$ where $n \geq m$, we examine the left-hand $m \times m$ portion of this matrix which we denote $F_{\square}$. Each decaying eigenvalue of the full operator F that can be identified at this stage in the calculation is part of the spectrum of $F_{\square}$, although the reverse is not true. We therefore use the spectrum of $F_{\square}$ as a set of candidate eigenvalues, and for each sufficiently-small candidate λ (based on a user-defined threshold) find the linearly dependent subspace using a QR decomposition of $F_{partial} - \lambda I_{partial}$, where $I_{partial}$ is another $m \times n$ matrix having 1s on the diagonal and 0s elsewhere.

Each constraint is the result of a linear relationship between the update rules appearing after a certain number of time steps, implying that the same linear relationship in the respective product basis variables appears one time step later. The general form of a linear dependency D beginning at time step t_c is:

$$D = c_\alpha x_\alpha + c_\beta x_\beta + \ldots \underset{t \geq t_c}{=} 0$$

where the subscript under the equals sign indicates the range of simulation time over which linear dependency D is valid. There are several ways in which constraints derived from this dependency could help simplify the calculation, but we must be careful that the application of a constraint should not undo the work of a prior constraint, in order to guarantee that the algorithm eventually terminates. Furthermore, the contraint algorithm should have a self-consistent way of removing a variable with two or more simultaneous constraints. Here we give two constraint algorithms that help to simplify late time analyses in the cases of probabilistic and deterministic Boolean models respectively.

Algorithm 5 (Equation reduction method 1)**.** *Given linear dependency D, we choose the lowest-index variable x_ϕ (which may be $x_\emptyset = 1$) based on a binary representation of its indices ϕ as in Appendix 1. Next, we remove that variable from all current and future time evolution equations in F by simple substitution: $x_\phi \to -(c_\alpha/c_\phi)x_\alpha - (c_\beta/c_\phi)x_\beta - \cdots$. Constraining a time evolution equation valid from time t_e using this formula yields a different time evolution equation valid for $t \geq \max(t_e, t_c)$.*

Proof. The substitution is justified for $t \geq t_c$, so the resulting time evolution equation is accurate after time t_c. A given constraint either removes a variable (if the constraint polynomial contains only one term), or else replaces a variable with higher-index variables. Each replacement with higher-index variables increases the index number of each constrained term by at least 1, and since the maximum index number is 2^N, the process of applying constraints to a given term in the equations eventually completes. Each constraint application permanently removes one variable from the finite 2^N-sized variable space, so the process of constraining the equations also completes, which is only possible if there are no more fast-decaying modes. Thus this process eventually removes the fast-decaying eigenspace, while preserving the slowly-decaying/persistent eigenspace. $\square$

We use equation reduction method 1 for PBNs and asynchronous networks as equation reduction method 2 (see Algorithm 6 below) does not apply to these networks. Equation reduction method 1 is relatively inefficient since it removes only one variable per constraint. There is also some indeterminism: if two constraints apply to a given term in an equation, we can choose only one of the two constraints to apply.

Here we demonstrate equation reduction method 1 by using it to find the attractors of the deterministic network from Example 1 in the main text.

Extension of Example 1

After solving Eqs. 1.1-1.6 in Example 1, we find that they contain a single linear dependency: $f_{AB} - f_{ABC} = 0$. Since the network is deterministic, this dependency guarantees that after the first time step (i.e. $t \geq 2$), x_{AB} will equal x_{ABC}, so we write $x_{AB} \underset{t \geq 2}{=} x_{ABC}$. We use this fact to remove x_{AB}, giving a new set of steady state equations:

$$\begin{aligned} f_A &= 1 - x_B \\ f_B &= x_{AC} \\ f_\emptyset &= 1 \\ f_{AC} &\underset{t \geq 2}{=} x_A - x_{ABC} \\ f_{ABC} &= x_{AC} - x_{ABC}. \end{aligned} \qquad (1.4')$$

Our new set of equations has the same non-zero eigenspace as the original set (1.1-1.6). However, the new equations lack the null eigenspace because we removed the only linear dependency. The 5×5 linear system has 5 eigenvalues, all having phases that are multiples of $2\pi/5$, indicating that the sole attractor is a limit cycle with a period of 5 time steps. We acknowledge that in this case the constraint helps find the attractors, but does not simplify the calculation, since it only appears after the full time evolution operator F has been calculated.

Algorithm 6 (Equation reduction method 2)**.** *Given linear dependency D, we obtain a constraint from each*

variable x_ϕ whose indices ϕ have the property $\gamma \backslash \phi \neq \emptyset$ for every other term x_γ in the dependency. Constraining a time evolution equation of a deterministic model valid from time t_e involves constraining every variable x_ρ in that equation that is factorized by one of the x_ϕ, in the sense that $\phi \backslash \rho = \emptyset$. Application of the constraint takes $x_\rho \to -(c_\alpha/c_\phi)x_{\alpha\cup\rho} - (c_\beta/c_\phi)x_{\beta\cup\rho} - \cdots$, yielding an equation that is valid for $t \geq \max(t_e, t_c)$.

Proof. For $t \geq \max(t_e, t_c)$, both the original time evolution equation and the linear dependency equation are valid, implying that $x_\phi = -(c_\alpha/c_\phi)x_\alpha - (c_\beta/c_\phi)x_\beta - \cdots$. For deterministic models, $x_\rho = x_\phi \cdot x_\rho$ since $\phi \subseteq \rho$ and indices can be freely duplicated in deterministic models. Substituting the two formulas gives the constraint rule. Since each constraint application either removes x_ϕ or replaces it with variables containing more indices, we can use the same arguments given for equation reduction method 1 to show that the method eventually removes the fast-decaying eigenspace while leaving correct time evolution equations for the slower-decaying modes. □

Equation reduction method 2 is considerably more aggressive than equation reduction method 1, because each constraint removes not only one or more variables involved in the constraint, but also any variables that are factorized by those constraints. As such, a dependency involving a variable with a lower index can exclude a significant fraction of the variable space. We demonstrate equation reduction method 2 using Example 2.

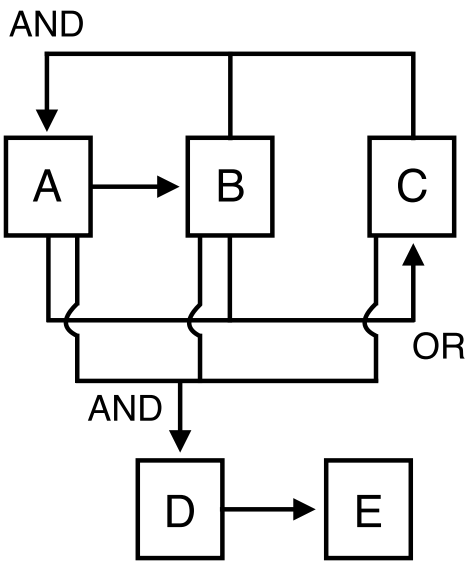

Figure 4. The network used in Example 2.

Example 2: applying constraints using equation reduction method 2

Suppose we want to find the late time behavior of Boolean variables A, C and E in the network from Figure 4. After two iterations of solving for time evolution functions we have:

$$f_A = x_{BC} \tag{2.1}$$
$$f_C = x_A + x_B - x_{AB} \tag{2.2}$$
$$f_E = x_D \tag{2.3}$$

$$f_B = x_A \tag{2.4}$$
$$f_D = x_{ABC} \tag{2.5}$$
$$f_{AB} = x_{ABC} \tag{2.6}$$
$$f_{BC} = x_A \tag{2.7}$$

At this point there are two linear dependencies: $f_{AB} = f_D$ and $f_{BC} = f_B$, implying that:

$$x_{AB} \underset{t\geq 2}{=} x_D \tag{C1}$$
$$x_D \underset{t\geq 2}{=} x_{AB} \tag{C2}$$
$$x_B \underset{t\geq 2}{=} x_{BC}. \tag{C3}$$

Since we are only interested in the late time behavior, we will use constraints C1-C3 to simplify the equations. Note that if we were to retain, for example, x_{ABC}, it would be affected by the constraints involving variables x_{AB}, x_B, and x_{BC}, thus it would not be possible to enforce all of the constraints by substitution because there is only one B-index on x_{ABC} to substitute for. However, we can simultaneously enforce all constraints by *multiplying* them:

$$\begin{aligned} x_{ABC} &= x_{AB} \cdot x_B \cdot x_{BC} \cdot x_{ABC} \\ &\underset{t\geq 2}{=} x_D \cdot x_{BC} \cdot x_B \cdot x_{ABC} \\ &= x_{ABCD}. \end{aligned}$$

Viewed another way, constraint C1 attaches indices of AB to every variable containing a D, and an index of D to each variable with indices of AB. Constraint C2 adds an index of C to every variable with an index of B.

After constraining our system and removing the time evolution equations of the removed variables, we have:

$$f_A = x_{BC}$$

$$f_C \underset{t\geq 2}{=} x_A + x_{BC} - x_{ABCD} \quad (2.2')$$

$$f_E \underset{t\geq 2}{=} x_{ABCD} \quad (2.3')$$

$$f_{BC} = x_A.$$

Our new equations require us to solve for another variable (while applying the constraints):

$$f_{ABCD} \underset{t\geq 2}{=} x_{ABCD}. \quad (2.8)$$

The system is now closed (5 equations involving 5 variables), so if our goal is to produce a simulation (valid for $t \geq 2$) then we are done. However, if our objective is to find the attractors, then we must remove another dependency that was unmasked by the last equation: $f_C \underset{t\geq 2}{=} f_{BC} + f_A - f_E$, which implies three useful constraints:

$$x_A \underset{t\geq 3}{=} x_C + x_E - x_{BC} \quad (C4)$$

$$x_C \underset{t\geq 3}{=} x_A - x_E + x_{BC} \quad (C5)$$

$$x_E \underset{t\geq 3}{=} x_A - x_C + x_{BC}. \quad (C6)$$

Each constraint reduces the size of the variable space. For example, constraint C4 removes all variables containing index A with no C, E, or BC. We did not bother to solve for x_{BC} in terms of x_A, x_B and x_C because that gives us a useless constraint: multiplying a variable having BC indices by x_C does not change that variable.

After applying constraints C4-C6, we obtain:

$$f_{BC} \underset{t\geq 3}{=} x_{AC} + x_{AE} - x_{ABCD} \quad (2.7')$$

$$f_{ABCD} \underset{t\geq 2}{=} x_{ABCD}$$

$$f_{AC} = x_{BC} \quad (2.9)$$

$$f_{AE} \underset{t\geq 2}{=} x_{ABCD} \quad (2.10)$$

$$f_{CE} \underset{t\geq 2}{=} x_{ABCD} \quad (2.11)$$

$$f_{BCE} \underset{t\geq 2}{=} x_{ABCD}. \quad (2.12)$$

This produces new dependencies: $f_{AE} \underset{t\geq 2}{=} f_{ABCD}$ and $f_{CE} \underset{t\geq 2}{=} f_{ABCD}$, implying that

$$x_{AE} \underset{t\geq 3}{=} x_{ABCD} \quad (C7)$$

$$x_{ABCD} \underset{t\geq 3}{=} x_{AE} \quad (C8)$$

$$x_{CE} \underset{t\geq 3}{=} x_{ABCD}. \quad (C9)$$

After applying constraints C7-C9, our example ends with the following closed system of dynamical equations:

$$f_{BC} \underset{t\geq 3}{=} x_{AC} \quad (2.7'')$$

$$f_{ABCDE} \underset{t\geq 3}{=} x_{ABCDE} \quad (2.13)$$

$$f_{AC} = x_{BC}.$$

The attractor is always reached at or before time step 3. The constraint equations (C1-C9) map our variables of interest (x_A, x_C, and x_E) to linear combinations of variables in the final system. For example, x_A is mapped by constraint C4 to $x_{AC} + x_{AE} - x_{ABC}$, then mapped using constraints C1, C7, and C8 to x_{AC}, whose dynamics are given by the final time evolution equations. The eigenvalues of this final system are -1, 1 and 1, implying a steady state along with a limit cycle having a period of 2 time steps.

When using equation reduction method 2, the order in which we calculate the time evolution of new variables and remove dependencies greatly influences the complexity of the calculation. One general rule is that constraints on the lowest-index variables tend to be most helpful for deterministic networks, because they factorize the largest part of the product variable space. Following this rule of thumb, our implementation solves the time evolution equations for only those variables having the fewest indices between each search for new dependencies. Additionally, our code solves for low-index factors of new variables even if they are not directly involved in earlier equations in hopes that they will produce helpful constraints. In most tests, this prioritization method speeds up the calculations.

Equation reduction method 2 is susceptible to numerical problems that fall into two categories: 1) polynomial coefficients can become very large, and 2) gradual erosion of numerical precision can blur the distinction between zero and nonzero terms. Our code monitors both sources of error; in the latter case, by tracking the floating-point error in each step of the calculation. When either type of calculation error exceeds defined bounds, our method exits with an error message.

The reason polynomial coefficients can become large is that multiplication by constraints can produce several identical terms that add together. For example, the constraint $x_1 = x_2 + x_3$ takes $x_{123} \to 2x_{123}$. Typically the variable being multiplied would be found to be zero later in the calculation (so having a coefficient of 2 is not an error in the math), but if many such constraints are applied and the coefficient grows exponentially, then the numerics will eventually fail. We have included a filter that can usually identify these outlier coefficients and remove them using the rule that all individual (not population-averaged) product variables are either 0 or 1 at all time steps. For example, if $f_4 = 2x_{123}$, we can reason that if f_4 is either 0 or 1, then x_{123} cannot be 1 and therefore we have a new constraint $x_{123} = 0$, which simplifies $f_4 \to 0$. In general, we identify these removable terms using the heuristic that if a large coefficient multiplying a combination of integer-weighted variables is greater than the sum of all other coefficients plus one, then that combination of variables (which is an integer for a homogeneous population) must be zero.

Our equation reduction method 2 was tested by running 10^4 experiments comparing Monte Carlo to our product basis method. Each experiment used a unique randomly-generated 10-node Boolean model having 1-4 inputs per node as before, and involved a population of 100 individuals starting in different random states. The comparison was between a) the dynamics of the product basis equations using a random amount of equation reduction (which determines the earliest time point at which the product basis simulation is valid), and b) the population average of the individual simulations. In each case the product basis simulation exactly reproduced the average of the Monte Carlo simulations. These networks were small, but still provided a stringent test of the numerics, as they could theoretically involve linear systems of equations up to size $(2^{10})^2$. During this evaluation, our program also generated additional networks whose numerical error in the polynomial coefficients overstepped a numerical threshold of 10^{-6} and were thus omitted from the Monte Carlo comparison; overall 18.5% of tests generated were discarded.

Appendix 3: algorithm and code

Pseudocode is given in Algorithm 1. For convenience, we sometimes write the solved update rules F as a set denoted F_{set}. The full code is available at: `https://github.com/CostelloLab/ProductBasis`.

Algorithm 1 build time evolution equations in F

Initialize set of variables to variables of interest: $X \leftarrow \{x_\sigma, x_\zeta, \dots\}$
Initialize set of variable update rules: $F_{set} \leftarrow \emptyset$
Initialize set of constraints: $C \leftarrow \emptyset$
Initialize simulation start time: $t_{sim} \leftarrow 1$
Initialize set of equation start times of F: $T_F \leftarrow \emptyset$
Initialize set of constraint start times: $T_C \leftarrow \emptyset$

while $\{\alpha \mid x_\alpha \in X, f_\alpha \notin F_{set}\}$ is not empty **do**

    % Reduce equations if necessary
    if $size(F_{set}) > equation\_reduction\_threshold$ **then**
        $F_\square \leftarrow$ matrix of terms in x_α having corresponding $f_\alpha \in F_{set}$
        $\lambda \leftarrow$ set of eigenvalues of $F_\square^T$
        for each $\lambda_i \in \lambda$ **do**
            $G \leftarrow F - \lambda_i [I\ 0]$
            $R \leftarrow$ upper-triangular matrix from QRdecomposition(G)
            $V \leftarrow$ submatrix of G consisting of rows a for which $|R_{aa}| < \epsilon$
            $W \leftarrow$ submatrix of G consisting of rows b for which $|R_{bb}| \geq \epsilon$
            $D \leftarrow$ set of linear dependencies found by solving $D \cdot V = W$
            if $V \neq \emptyset$ **then**
                $t_{sim} \leftarrow \min(t_{sim}, \min(T_F(x_k \in D)) + \max(1, \mathrm{ceiling}(\log \epsilon_{decay} / \log |\lambda_i|)))$
                break
            end if
        end for
        $D \leftarrow \{d_i \in D$ whose number of terms $\leq 2 \cdot \min_i$(number of terms in d_i)$\}$
        Sort elements of D in increasing order of number of terms
        for each $d_i \in D$ **do**
            NewConstraints(d_i, t_{sim})
        end for
        Sort elements of C in increasing order of number of terms
    end if

    % Add new equations
    $X \leftarrow X \cup$ lowest-index factors of X
    Sort elements of X in increasing order of number of terms
    for each $\{\alpha \mid x_\alpha \in X, f_\alpha \notin F_{set}\}$ **do**
        $f_\alpha \leftarrow 1$
        for each $i \in \alpha$ **do**
            if f is a discrete update rule **then**
                $f_\alpha \leftarrow f_\alpha \cdot f_i$
            else
                $f_\alpha \leftarrow f_\alpha + f_i \cdot \prod_{(j \in \alpha) \neq i} x_j$
            end if
        end for
        $F_{set} \leftarrow F_{set} \cup$ Constrain($f_\alpha, 1$)
    end for

end while

function NEWCONSTRAINTS(d, t_c)
if $d \leftarrow$ REMOVEOVERSIZECOEFFICIENTS$(d/\text{minCoef}(d))$ adds no new constraint **then**
for each $x_\alpha \in d$ for which $\alpha \not\subseteq \beta$ for any another term $x_\beta \in d$ **do**
$RHS_\alpha \leftarrow$ solve $d = 0$ for x_α
if $\{x_\alpha\} \neq$ CONSTRAIN$(\{RHS_\alpha\}, t_c)$ **then**
for each prior constraint c_γ of the form $x_\gamma = RHS_\gamma$ **do**
if $\alpha \subseteq \gamma$ **and** $x_\gamma \cdot RHS_\alpha = RHS_\gamma$ **then**
$C \leftarrow C \setminus \{c_\gamma\}$
end if
end for
$C \leftarrow C \cup \{x_\alpha = RHS_\alpha\}$
for each $x_\psi \mid \alpha \subseteq \psi$ **do**
$poly_\psi \leftarrow$ CONSTRAIN(x_ψ, t_c)
$X \leftarrow X \cup$ unsolved x_β in $poly_\psi$
$A \leftarrow \{a_l =$ nonzero coefficients of x_ψ in $F\}$
$F \leftarrow F + A \cdot (poly_\psi - x_\psi)$
$(t_{f_l}$ for all l such that $a_l \neq 0) \leftarrow t_c$
end for
end if
end for
end if
end function

function CONSTRAIN$(poly, t_c)$
while $\exists$ unconstrained terms in *poly* **do**
for each constraint beginning at time t_c having the form $x_\phi = RHS_\phi$ **do**
% Multiply all factored variables by the constraint
for each unconstrained variable x_α with coefficient a_α in *poly* **do**
if $\phi \subseteq \alpha$ **then**
$poly_{new} \leftarrow x_\alpha \cdot RHS_\phi$
if $x_\alpha \not\subset poly_{new}$ **then**
$poly \leftarrow poly - a_\alpha \cdot x_\alpha + poly_{new}$
$t_{f_i} \leftarrow t_c$
end if
end if
end for
poly $\leftarrow$ REMOVEOVERSIZECOEFFICIENTS(*poly*)
end for
end while
return *poly*
end function

% Check for overlarge coefficients
function REMOVEOVERSIZECOEFFICIENTS(*poly*)
while $\exists$ unchecked coefficients in *poly* **do**
for each coefficient a_i in *poly* **do**
$n_j = \text{round}(a_j/a_i)$ for all coefficients $j \neq i$
if $|a_i| > \sum_j |a_j - n_j a_i|$ **then**
NEWCONSTRAINTS(n_j, t_{sim})
$poly \leftarrow poly - n_j a_i$ for all coefficients $j \neq i$
break
end if
end for
end while
return *poly*
end function
